# Regulation of Ca^2+^/Calmodulin-Dependent Protein Kinase II Signaling within Hippocampal Glutamatergic Postsynapses during Flurazepam Withdrawal

**DOI:** 10.1155/2012/405926

**Published:** 2012-07-05

**Authors:** Damien E. Earl, Paromita Das, William T. Gunning, Elizabeth I. Tietz

**Affiliations:** ^1^Department of Physiology and Pharmacology, The University of Toledo College of Medicine, Health Science Campus, 3000 Arlington Avenue, Mailstop 1008, Toledo, OH 43614, USA; ^2^Department of Pathology, The University of Toledo College of Medicine, Health Science Campus, Toledo, OH 43614, USA; ^3^Department of Neurosciences, The University of Toledo College of Medicine, Health Science Campus, 3000 Arlington Avenue, Mailstop 1008, Toledo, OH 43614, USA

## Abstract

Cessation of one-week oral administration of the benzodiazepine flurazepam (FZP) to rats results in withdrawal anxiety after 1 day of withdrawal. FZP withdrawal is correlated with synaptic incorporation of homomeric GluA1-containing **α**-amino-3-hydroxy-5-methylisoxazole-4-propionic acid receptors (AMPARs) in the proximal stratum radiatum of CA1 neurons. After 2 days of withdrawal, Ca^2+^/calmodulin-dependent protein kinase II (CaMKII) phosphorylates GluA1 subunits at Ser^831^, increasing channel conductance. Secondary to AMPAR potentiation, GluN2B-containing N-methyl-D-aspartate receptors (NMDARs), known binding partners of CaMKII, are selectively removed from the postsynaptic density (PSD). While activation of synaptic CaMKII is known to involve translocation to the PSD, CaMKII bound to NMDARs may be removed from the PSD. To distinguish these possibilities, the current studies used postembedding immunogold electron microscopy to investigate alterations in CaMKII signaling at CA1 stratum radiatum synapses after 2 days of FZP withdrawal. These studies revealed decreased total, but not autophosphorylated (Thr^286^) CaMKII**α** expression in CA1 PSDs. The removal of CaMKII-GluN2B complexes from the PSD during drug withdrawal may serve as a homeostatic mechanism to limit AMPAR-mediated CA1 neuron hyperexcitability and benzodiazepine withdrawal anxiety.

## 1. Introduction

Ca^2+^/calmodulin-dependent protein kinase II (CaMKII) is a dodecameric holoenzyme composed of a combination of four enzyme isoforms (*α*, *β*, *γ*, or *δ*), though *α* and *β* are the primary isoforms expressed in neurons [1]. CaMKII molecules within the holoenzyme autophosphorylate adjacent enzymes at Thr^286^ (or Thr^287^ in non-*α* isoforms) during persistent activation by Ca^2+^/CaM [2], leading to autonomous, Ca^2+^-independent CaMKII activity [3]. This feature allows CaMKII to maintain a molecular memory of recent neuronal activity and is a critical component of activity-dependent long-term potentiation (LTP), a form of synaptic plasticity in hippocampal CA1 neurons thought to underlie learning and memory [4]. Molecular memory is also conferred upon CaMKII by its ability to translocate to the postsynaptic density (PSD) from intracellular synaptic compartments following its activation by Ca^2+^/CaM and its subsequent autophosphorylation [5, 6]. During LTP, CaMKII potentiates *α*-amino-3-hydroxy-5-methyl-4-isoxazolepropionic acid receptors (AMPARs) by increasing both receptor number [7] and single channel conductance via GluA1 subunit AMPAR phosphorylation [8]. CaMKII activation during LTP is dependent on NMDAR-mediated Ca^2+^ influx, whereas L-type voltage-gated Ca^2+^ channels (L-VGCCs) may activate CaMKII in nucleus accumbens following chronic cocaine exposure [9, 10]. In addition, while L-VGCCs are potentiated in CA1 neurons after withdrawal from benzodiazepines and may initiate CaMKII-mediated potentiation of AMPARs [11–13], the mechanisms of CaMKII signaling during benzodiazepine withdrawal remain unknown.

While benzodiazepines are clinically useful anxiolytics, sedative-hypnotics, and anticonvulsants, long-term treatment can result in the development of physical dependence manifested by withdrawal symptoms such as anxiety, insomnia, and, rarely, seizures, limiting their clinical benefit [14]. The clinical effects of benzodiazepines are mediated by allosteric enhancement of inhibitory currents through *γ*-aminobutyric acid type A receptors (GABA_A_Rs), whereas current evidence suggests that benzodiazepine withdrawal symptoms are associated with potentiation of AMPAR-mediated glutamatergic transmission [11, 15–18]. Similar to mechanisms of LTP in CA1 neurons, a temporal pattern of AMPAR potentiation was observed in rats withdrawn from the benzodiazepine, FZP. After 1 day of FZP withdrawal, AMPAR current amplitude was increased due to postsynaptic density (PSD) incorporation of GluA1 homomeric AMPARs [13, 18, 33]. AMPAR conductance increased after 2 days of FZP withdrawal associated with Ser^831^ phosphorylation of GluA1 subunits by CaMKII [13, 19]. Interestingly, a reduction of NMDAR current was also observed 2 days after withdrawal, mediated by removal of GluN1/GluN2B-containing NMDARs from CA1 PSDs [20], which normalized total excitatory current output in CA1 neurons and precluded expression of withdrawal anxiety [16, 19, 21].

The GluN2B subunit is an important binding partner of CaMKII*α* [6, 22], and it is feasible that removal of GluN2B-containing NMDARs may alter CaMKII signaling during FZP withdrawal. Prior studies in our laboratory detected increased total CaMKII*α* expression in PSD-enriched CA1 homogenates from 2-day FZP-withdrawn rats without a concomitant increase in autophosphorylated Thr^286^-CaMKII (pCaMKII) [13]. Although the fractionated homogenate was enriched in PSDs, other non-PSD subcellular compartments were also present in the homogenate [18]; thus, this was a less accurate measure of total and autophosphorylated CaMKII expression within the synapse.

To gain a more precise understanding of the potential alterations in CaMKII signaling during FZP withdrawal, the current studies utilized quantitative postembedding immunogold electron microscopy (EM) to assess localization of total and Thr^286^ autophosphorylated CaMKII within asymmetric synapses of CA1 *stratum radiatum* (SR) in tissues from 2-day FZP-withdrawn and matched control rats. The findings revealed a significant reduction in total, but not autophosphorylated CaMKII*α* within CA1 PSDs of 2-day FZP-withdrawn rats. The loss of CaMKII*α* from PSDs is consistent with removal of GluN2B NMDARs after 2 days of FZP withdrawal and may serve as a homeostatic mechanism to limit AMPAR potentiation and FZP withdrawal symptoms.

## 2. Materials and Methods

### 2.1. Long-Term FZP Treatment

All procedures involving the use of animals were performed in compliance with The University of Toledo College of Medicine Institutional Animal Care and Use Committee (IACUC) and National Institutes of Health guidelines. One-week oral treatment of rats with the relatively water-soluble benzodiazepine FZP was as described previously [16]. Briefly, male Sprague-Dawley rats (P22-25, Harlan, Indianapolis, IN) were acclimated 2–4 days to 0.02% saccharin water. Rats were then offered saccharin water containing FZP (pH 5.8) as the sole drinking source. The FZP concentration was periodically adjusted based on rat's body weight and the volume consumed to yield a dosage of 100 mg/kg/day for 3 days, then 150 mg/kg/day for 4 days. The final average daily FZP dose was always greater than 100 mg/kg, generally 125–130 mg/kg, with a goal of achieving a minimum average daily dose of 120 mg/kg [20]. Related to its oral bioavailability and the half-life (<12 hrs) of FZP and its major bioactive metabolites in rats [23], this treatment paradigm results in brain benzodiazepine levels of about 1.2 *μ*M measured in rat brain homogenates by radioreceptor assay [24]. After 1-week FZP treatment, rats were offered saccharin water for 1 or 2 days. FZP withdrawal consistently results in anxiety-like behavior after 1 day of withdrawal [11, 16], which can be masked or expressed on day 2 of withdrawal as a function of the appearance or reversal of NMDAR downregulation [16, 21]. Matched control rats were offered saccharin water for the same experimental period.

### 2.2. Antibodies Used for Postembedding Immunogold Electron Microscopic Analysis

Monoclonal anti-CaMKII*α* antibody (clone 6G9-2, Millipore, Billerica, MA, Cat. no. MAB8699) was generated using purified CaMKII*α* protein [25, 26]. This antibody detected a band at approximately 50 kDa, consistent with the molecular weight of CaMKII*α*, whereas no bands were observed in hippocampal lysates from knockout mice [27], confirming its specificity. A polyclonal anti-pCaMKII antibody which recognizes the epitope MHRQET(PO_4_)VDCLKKFN was from Promega (Madison, WI; Cat. no.: V1111). This antibody recognizes phosphorylated Thr^286/287^ probably on all CaMKII isoforms [28, 29]. Labeling using this antibody was substantially reduced when used in immunocytochemical studies of visual cortex from mice with CaMKII*α* Thr^286^ mutated to alanine [30]. Preembedding immunogold pCaMKII labeling using this antibody also revealed increased postsynaptic labeling in hippocampal cultures exposed to NMDA [31]. To test the specificity of these two antibodies, controls used in the current studies included omitting the primary antibody, revealing labeling with each gold-conjugated secondary antibody alone. Additionally, cross-reaction of the secondary antibodies was tested by replacement of each primary antibody with the other.

### 2.3. Postembedding Immunogold Electron Microscopy

The ultrathin cut sections used in the current studies were cut from the same tissues as those used in a previous study of GluN1 and GluN2A/B subunits at CA1 neuron asymmetric postsynapses [20]. The transcardial fixation, cryosubstitution, and postembedding immunogold EM labeling used for these tissues were described previously. Briefly, isoflurane anesthetized rats were transcardially perfused with an oxygenated vascular rinse followed by 4% paraformaldehyde and 0.5% glutaraldehyde. Hippocampal slices (200 or 500 *μ*m) were slam-frozen (−190°C, Leica EM CPC, Bannockburn, IL), cryosubstituted, and flat-embedded in lowicryl resin. Ultrathin CA1 sections (80 nm) were collected on nickel grids, equilibrated in Tris-buffered saline with 0.1% Triton X-100 (TBST, pH 7.6), quenched in 1% NaBH_4_ and 50 mM glycine, blocked in 10% normal goat serum (NGS), incubated in primary antibodies (1 : 20 anti-CaMKII*α* and/or 1 : 10 anti-pCaMKII with 1% NGS) 2 hr at room temperature, then overnight at 4°C. Tissues were switched to pH 8.2 TBST, incubated in 0.5% poly(ethylene) glycol (PEG), then in secondary antibody containing 0.5% PEG (1 : 25 goat anti-rabbit IgG conjugated to 10 nm gold and/or 1 : 25 goat anti-mouse IgG conjugated to 15 nm gold, BBInternational, UK) for 1.5 hr. Sections were counterstained with 5% uranyl acetate and Reynold's lead citrate.

The CA1 proximal SR region of reacted tissues was scanned using a Phillips CM10 PW6020 transmission electron microscope to randomly identify profiles of asymmetric synapses, which are presumed glutamatergic synapses because axonal projections containing glutamate largely predominate over projections containing other excitatory neurotransmitters in CA1 neurons [32]. Images were captured at 52,000X magnification. The negatives were developed and scanned, then immunogold labeling was measured using Image Pro Plus software (Media Cybernetics, Inc., Bethesda, MD) as previously described [20]. Pre- and postsynaptic immunogold particles were binned into distances out to 300 nm perpendicular to the cleft surface of the PSD. Immunogold particles were also assigned to PSD, active zone, perisynaptic, membrane, or intracellular compartments. Particles within 20 nm (approximate size of the immunogold antibody complex) of the PSD were counted as PSD labeling as described previously [33]; particles within 20 nm of the presynaptic membrane, but not within 20 nm of the PSD membrane, were considered within the active zone; particles within 20 nm of the membrane were considered membrane-bound; postsynaptic membrane-bound particles within 100 nm lateral to the PSD were considered perisynaptic [33]. All procedures and measurements were performed with the experimenter blinded to the experimental groups.

### 2.4. Statistical Analyses

The percentage of various synaptic compartments with immunogold labeling and the mean number of immunogold particles were compared between control and FZP-withdrawn groups by Student's *t*-test. Presynaptically and postsynaptically binned immunogold particles were analyzed by two-way repeated measures (mixed-model) ANOVA with post hoc comparisons between control and FZP-withdrawn groups within each bin using Bonferroni's Multiple Comparison test. The relative frequency of the number of immunogold particles per PSD (0 to 5) was compared between control and FZP-withdrawn groups by Mann-Whitney *U*-test. Data were statistically analyzed and graphs generated using Prism 4.0 software (GraphPad Software Inc., San Diego, CA). Values are reported as mean ± SEM and were considered significantly different if the *P* value was less than or equal to 0.05.

## 3. Results

### 3.1. CaMKII*α* Expression, but not Thr^286^ Autophosphorylation, Is Reduced in FZP-Withdrawn CA1 Asymmetric Postsynapses

Prior evidence suggests that in rats withdrawn from FZP for 2 days, CaMKII may be activated by Ca^2+^ influx through L-VGCCs to phosphorylate GluA1 subunits at Ser^831^ increasing AMPAR conductance [11, 13, 19, 34]. To further evaluate the nature of CaMKII activation and its postsynaptic localization, ultrathin sections of the CA1 region were colabeled with anti-CaMKII*α* and anti-autophosphorylated Thr^286/287^ CaMKII*α*/*β* (anti-pCaMKII) antibodies and distinguished using secondary antibodies conjugated to 15 or 10 nm gold, respectively. Dual labeling was assessed in asymmetric synapses in the proximal dendritic SR region, where increased PSD expression of the GluA1 AMPAR subunit was previously detected [33]. Decreased PSD expression of GluN1 and GluN2B NMDAR subunits was also found in the same tissues used in the current studies [20]. Similar levels of labeling were observed in tissues reacted with CaMKII or pCaMKII antibodies alone (data not shown) in comparison to antibodies combined as a cocktail, suggesting that the antibodies did not sterically interfere with each other during the dual reaction. Omission of the anti-CaMKII*α* antibody yielded 0.01 15 nm immunogold particles/bouton, 0.10 particles/spine, and 0.01 particles/PSD in 69 asymmetric synaptic profiles compared with 0.62 ± 0.23 particles/bouton, 1.41 ± 0.36 particles/spine, and 0.48 ± 0.15 particles/PSD on average in control tissues. Cross-reaction of the goat anti-mouse secondary antibody conjugated to 15 nm gold with the opposite (rabbit anti-pCaMKII) primary was tested by replacement of the monoclonal anti-CaMKII*α* antibody with the polyclonal anti-pCaMKII antibody, which yielded no pre- or postsynaptic 15 nm immunogold particles within 60 randomly selected asymmetric synaptic profiles. Omission of the polyclonal anti-pCaMKII antibody yielded 0.30 10 nm immunogold particles/bouton, 0.24 particles/spine, and 0.06 particles/PSD in 63 asymmetric synaptic profiles compared with 1.48 ± 0.55 particles/bouton, 1.06 ± 0.45 particles/spine, and 0.41 ± 0.13 particles/PSD on average in control tissues. Cross-reaction of the goat anti-rabbit secondary antibody conjugated to 10 nm gold with the opposite (mouse anti-CaMKII*α*) primary was tested by replacement of the polyclonal anti-pCaMKII antibody with the monoclonal anti-CaMKII*α* antibody, which yielded no pre- or postsynaptic 10 nm immunogold particles within 60 randomly selected asymmetric synaptic profiles. Taken together, these results support specific labeling by each primary antibody and no cross-reaction of the secondary antibodies with the opposite primary antibody.

**Figure 1:**
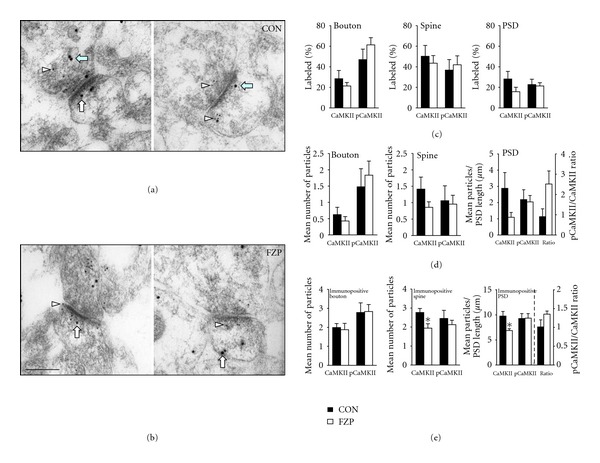
Images of asymmetric synapses in the CA1 SR of control (a) and 2-day FZP-withdrawn (b) rats colabeled with anti-CaMKII*α* (1 : 20, recognized with 15 nm gold, arrows) and anti-pCaMKII antibodies (1 : 10, recognized with 10 nm gold, arrowheads). CaMKII and pCaMKII immunogold particles were observed pre- and postsynaptically, both intracellularly and associated with the membrane and PSD. Scale bar = 0.2 *μ*m. (c) There was no significant change in the percent of boutons, spines, or PSDs labeled with either CaMKII or pCaMKII (*P* > 0.05 in each case). (d) There was a nonsignificant decrease in CaMKII labeling in spines (CON: 1.41 ± 0.36 particles, *n* = 5; FZP: 0.86 ± 0.17 particles, *n* = 5; *P* = 0.20), as well as decreased CaMKII immunogold particles/PSD length (CON: 2.88 ± 0.97 particles/*μ*m, *n* = 5; FZP: 1.10 ± 0.29 particles/*μ*m, *n* = 5; *P* = 0.12). However, without an alteration in pCaMKII labeling there was no change in the pCaMKII/CaMKII ratio in PSDs of 2-day FZP-withdrawn rats (CON: 1.14 ± 0.48, *n* = 5; FZP: 2.52 ± 0.64, *n* = 5; *P* = 0.12). (e) Analysis of immunopositive compartments (≥1 immunogold particle) revealed a significant decrease in CaMKII labeling in immunopositive spines (CON: 2.77 ± 0.22 particles, *n* = 5; FZP: 1.94 ± 0.23 particles, *n* = 5; *P* = 0.03) and PSDs (CON: 9.77 ± 0.92 particles/*μ*m; FZP: 6.88 ± 0.37 particles/*μ*m; *P* = 0.02) from 2-day FZP-withdrawn rats. However, the ratio of pCaMKII/CaMKII within the PSD was not significantly altered (CON: 1.01 ± 0.19, *n* = 5; FZP: 1.35 ± 0.09, *n* = 5; *P* = 0.14).

Figure 1 illustrates dual labeling observed in asymmetric synapses in tissues from control (A) and FZP-withdrawn (B) rats. Immunogold particles were counted within 300 nm pre- and postsynaptic to the cleft surface of the PSD. There was no significant change in the percentage of boutons, spines, or PSDs labeled with CaMKII or pCaMKII antibodies (Figure 1(c), Tables 1 and 2, resp.). Interestingly, a nonsignificant trend towards a decreased mean number of CaMKII immunogold particles was observed in spines (39% decrease, *P* = 0.20) and PSDs (62% decrease, *P* = 0.12, Figure 1(d), Table 1), with no apparent change in the mean number of pCaMKII immunogold particles in boutons, spines, or PSDs (Figure 1(d), Table 2). When only positively labeled (≥1 immunogold particle) compartments were analyzed, a significant decrease in the number of CaMKII immunogold particles was measured in spines (30% decrease, *P* = 0.03) and PSDs (30% decrease, *P* = 0.02), but not boutons (Figure 1(e), Table 1). No alteration in pCaMKII labeling was observed in immunopositive synaptic compartments (Figure 1(e), Table 2). The significant reduction in CaMKII*α* without a change in pCaMKII resulted in an apparent, but nonsignificant increase in pCaMKII/CaMKII expression (33%, *P* = 0.14, Figure 1(e), right panel). Within non-PSD compartments, a significant decrease in the mean number of CaMKII immunogold particles and the percent of active zones labeled was observed in tissues from FZP-withdrawn rats, but no significant alterations in CaMKII or pCaMKII immunogold labeling were observed in pre- or postsynaptic membrane or intracellular compartments (Table 3).

**Figure 2:**
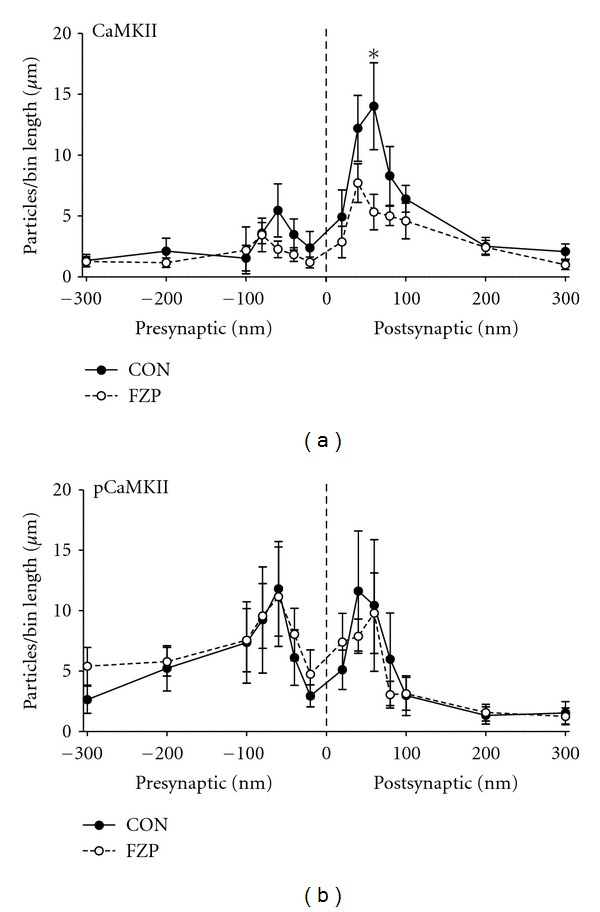
Pre- and postsynaptic distribution of CaMKII and pCaMKII immunogold particles in CA1 asymmetric synapses of control and FZP-withdrawn rats. (a) Analysis of CaMKII postsynaptic labeling revealed a significant interaction between experimental group and binned distance (*P* < 0.05, F(6,48) = 3.13, two-way repeated measures ANOVA) with significantly decreased CaMKII labeling 60 nm postsynaptic to the PSD membrane in FZP-withdrawn relative to control synapses (CON: 14.01 ± 3.57 particles/*μ*m, *n* = 5; FZP: 5.32 ± 1.45 particles/*μ*m, *n* = 5; *P* < 0.01, Bonferroni's posttest). In contrast, no significant interaction between treatment and binned distance was observed for presynaptic CaMKII (*P* = 0.34, F(6,48) = 1.16, two-way repeated measures ANOVA). (b) There was no significant interaction between experimental group and binned distance for pre- (*P* = 0.96, F(6,38) = 0.25) or postsynaptic pCaMKII labeling (*P* = 0.78, F(6,48) = 0.53, two-way repeated measures ANOVA).

**Figure 3:**
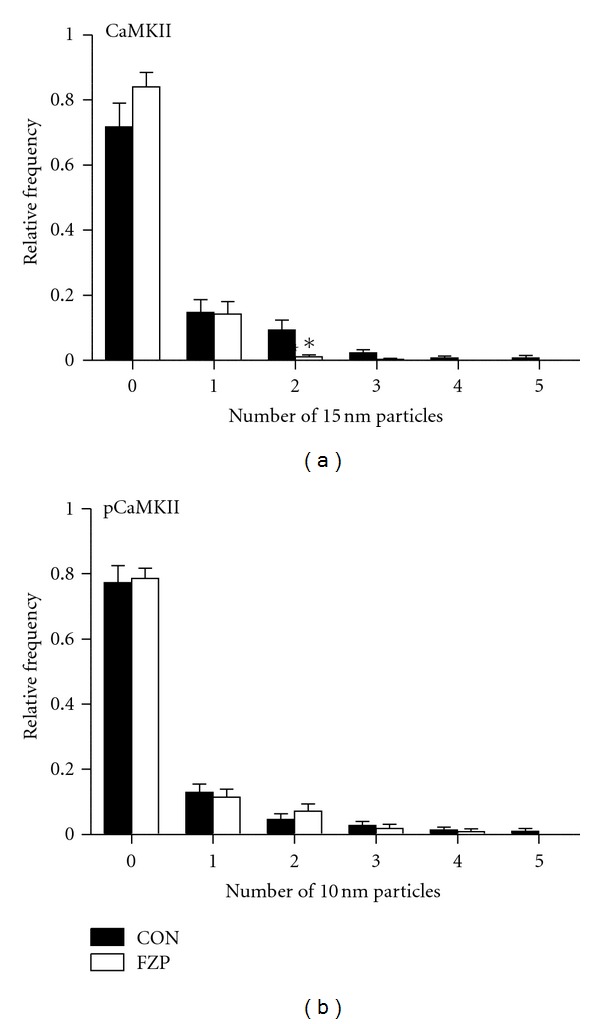
Distribution histograms of postsynaptic profiles containing different numbers of CaMKII (a) and pCaMKII (b) immunogold particles within the PSD. Histograms represent the average relative frequencies of synapses lacking (0 particles) CaMKII or pCaMKII immunogold particles or containing 1 to 5 immunogold particles. There was a significant decrease in the fraction of FZP-withdrawn synapses containing two CaMKII immunogold particles (CON: 0.094 ± 0.030, *n* = 5; FZP: 0.012 ± 0.005, *n* = 5; *P* < 0.05, Mann-Whitney *U*-test), whereas no significant difference was detected in the distribution of pCaMKII immunogold particles.

Figure 2 illustrates the mean number of CaMKII and pCaMKII immunogold particles in binned distances pre- and postsynaptic to the PSD membrane in both control and FZP-withdrawn rats. Analysis of binned CaMKII expression revealed a significant interaction between experimental group and binned distance with a significant 62% decrease in labeling 60 nm postsynaptic to the PSD membrane (Figure 2(a)). There was no significant interaction between experimental group and binned distance for presynaptic CaMKII expression or pCaMKII either pre- or postsynaptically. Because the average thickness of the CA1 neuron PSD previously measured in these same tissues was about 40 nm [20], and particles within 20 nm of the PSD were considered to represent PSD labeling, decreased CaMKII expression within the 60 nm postsynaptic bin corresponds to decreased PSD labeling, particularly near the intracellular surface of the PSD. Moreover, as illustrated by the distribution histograms of synapses containing different numbers of CaMKII or pCaMKII immunogold particles in Figure 3, there was a significant 87% decrease in the frequency of synapses containing 2 CaMKII immunogold particles in FZP-withdrawn tissues (*P* = 0.01). Collectively, these data support decreased CaMKII*α*, but not pCaMKII expression in PSDs within the CA1 proximal SR region of 2-day FZP-withdrawn rats.

## 4. Discussion

### 4.1. Decreased CaMKII Synaptic Localization during FZP Withdrawal

While enhanced L-VGCC Ca^2+^ current density likely induces CaMKII-mediated AMPAR potentiation during benzodiazepine withdrawal [11, 13, 34], the mechanisms of CaMKII signaling during withdrawal remain unknown. The goal of the current studies was to assess the pre- and postsynaptic localization of total CaMKII*α* and Thr^286/287^ autophosphorylated CaMKII. The primary finding was a loss of CaMKII*α* from the PSD, evidenced by the significantly lower number of CaMKII*α* immunogold particles within positively-labeled (≥1 immunogold particle) spines and PSDs and significantly lower relative frequency of PSDs labeled with 2 CaMKII*α* immunogold particles. There was also a significant decrease of CaMKII*α* immunogold particles within a binned distance 40 to 60 nm postsynaptic to the cleft surface of the PSD. This distance corresponds to the cytoplasmic surface of the PSD, because the average thickness of PSDs in these tissues is about 40 nm [20]. The 40 to 60 nm binned distance also corresponded to the highest density of CaMKII*α* immunogold particles. The predominant localization of CaMKII*α* at the cytoplasmic face of the PSD is consistent with a previous study, which indicated that CaMKII holoenzymes form stacked “towers” at the cytoplasmic surface of the PSD ranging from 20 to 60 nm in height, with 20 nm representing a single dodecameric holoenzyme [35]. A smaller peak of CaMKII*α* immunogold particle density was also observed at a binned distance 40 to 60 nm presynaptic to the cleft surface of the PSD. Interestingly, this bimodal distribution was considerably more exaggerated for pCaMKII, which had almost equivalent density of immunogold particles in the 40 to 60 nm bin, both pre- and postsynaptically. The latter finding suggests that the relative number of CaMKII molecules autophosphorylated at Thr^286/287^ is higher presynaptically in both control and FZP-withdrawn rats.

### 4.2. Implications of CaMKII Removal from CA1 PSDs during FZP Withdrawal

The reduction in total CaMKII*α* in immunogold-labeled tissues was surprising given that a prior study detected increased CaMKII*α* expression in the PSD-enriched subcellular fraction after 2 days of FZP withdrawal [13]. However, the Triton-insoluble PSD-enriched fraction contains other subcellular compartments in which CaMKII*α* expression may be increased. For example, CaMKII*α* is expressed in a heavy microsomal cytoskeletal compartment insoluble in 0.5% Triton X-100 [36]. It is possible that immunoblot analysis revealed increased CaMKII expression due to translocation to this cytoskeletal compartment, rather than to the PSD. Indeed, direct quantitation of CaMKII*α* expression using the quantitative immunogold EM technique in a blinded manner as in the current study is a more reliable method of assessing localization to the PSD than immunoblot analysis of the PSD-enriched subcellular fraction.

Prior studies established decreased expression of GluN1 and GluN2B subunits in CA1 PSDs after 2 days of FZP withdrawal [20, 21]. The loss of CaMKII*α* from CA1 asymmetric postsynapses corresponds to the loss of GluN1/GluN2B NMDARs observed in the prior EM studies using tissue sections from the same experimental groups used in the current studies [20]. Because GluN2B subunits were shown to bind CAMKII*α* [22, 37], postsynaptic removal of GluN2B-containing NMDARs may facilitate removal of CaMKII from the postsynaptic membrane. The removal of CaMKII-GluN2B complexes after 2 days of withdrawal could attenuate the enhanced AMPAR conductance due to GluA1 subunit phosphorylation at Ser^831^ [13], leading to restoration of the normal CA1 neuron excitability by day 4 of FZP withdrawal [16]. The reduction of CaMKII within CA1 PSDs may involve dissociation from or loss of postsynaptic proteins other than GluN2B, because CaMKII has other binding partners within the PSD [1]. However, CaMKII molecules combine to form dodecameric enzymes that can stack into towers of 2 to 3 holoenzymes [35]; thus, it is conceivable that each GluN2B-containing NMDAR lost from the PSD could actually remove 12 to 36 CaMKII*α* molecules.

### 4.3. CaMKII Activity during FZP Withdrawal: Role of Autophosphorylation and Binding Partners

The results also suggested an increased ratio of pCaMKII to CaMKII*α* immunogold particles within CA1 neuron PSDs after 2 days of FZP withdrawal. However, this result was not significant and the apparent change resulted primarily from the significant decrease in CaMKII*α* localized to the PSD without a change in pCaMKII PSD expression. This may indicate that during FZP withdrawal there was indeed an increased proportion of CaMKII molecules phosphorylated at Thr^286/287^, but it may also suggest that nonphosphorylated CaMKII*α* molecules were selectively lost from the PSD during FZP withdrawal, or conversely, that holoenzymes containing pCaMKII molecules remained selectively bound to PSD binding partners, consistent with prior studies [6, 22]. It is also possible that CaMKII undergoes autonomous activation by binding to the GluN2B subunit of NMDARs [22, 37], but whether the interaction of CaMKII with GluN2B-containing NMDARs is altered during FZP withdrawal remains to be determined.

It should be noted that the amino acid sequence surrounding the Thr^286/287^ autophosphorylation site is nearly identical in each CaMKII isoform; thus, the phosphospecific antibody used to detect Thr^286/287^ will recognize this epitope on all isoforms. In particular, CaMKII*β* is expressed at the second highest level in brain, with a CaMKII*α* to *β* ratio of about 3 : 1 in the forebrain [38, 39]. Unlike CaMKII*α*, CaMKII*β* contains an actin-binding segment that localizes holoenzymes to the actin cytoskeleton [1]. Autophosphorylation of CaMKII*β* causes heterooligomers to dissociate from actin enhancing translocation to the PSD [5]. Although there was no change in CaMKII*β* expression observed in CA1 PSD-enriched fractions from 2-day FZP-withdrawn rats [13], alterations in the expression of autophosphorylated CaMKII*β* cannot be ruled out.

Autophosphorylation results in autonomous CaMKII activity [3] and CaM trapping (1000-fold increase in CaM affinity) [40]. For many substrates such as the GluA1 subunit, autonomous CaMKII activity is only about 20% of maximal activity and CaMKII can be further stimulated by Ca^2+^/CaM, preventing complete uncoupling of CaMKII activation from neuronal activity [3]. Thus, it is also possible that increased CaMKII activity is maintained by sustained increases in Ca^2+^ concentrations near the PSD due to enhanced Ca^2+^ influx through L-VGCCs [34] and Ca^2+^-permeable GluA1 homomeric AMPARs [13, 16, 18]. This possibility is intriguing given that CaMKII also binds to L-VGCC *α*_1_ subunits, Ca_v_1.2 [41] and Ca_v_1.3 via a densin-180 interaction [42]. Although expression of the Ca_v_1.2, Ca_v_1.3, or *β*_3_ L-VGCC subunits was not altered in PSD-enriched homogenates from FZP-withdrawn rats [43], interaction of CaMKII with L-VGCCs may serve as an alternate locus for modulation of CaMKII activity rather than through its association with NMDARs. Phosphatase activity may also be reduced in FZP-withdrawn tissues, so that the same level of kinase activity is unopposed by a corresponding amount of phosphatase activity, as occurs during LTP in CA1 synapses [44]. Whether activity levels of specific phosphatases are altered during FZP withdrawal remains to be directly tested.

### 4.4. Conclusions and Implications

The current studies indicated a significant reduction in CaMKII*α* immunogold particles within CA1 PSDs without a change in the amount of CaMKII autophosphorylated at Thr^286^ after 2 days of FZP withdrawal. Loss of CaMKII*α* from CA1 PSDs after 2 days of FZP withdrawal correlates with the significant reduction in GluN1 and GluN2B immunogold labeling within CA1 PSDs previously observed in the same tissues [20]. Although other mechanisms may be involved, it is possible that complexes of CaMKII and GluN2B-containing NMDARs are removed from CA1 neuron PSDs as a compensatory mechanism to offset the observed enhancement of AMPAR-mediated CA1 neuron hyperexcitability [16, 18, 21]. Removal of synaptic GluN2B-containing NMDARs normalized total CA1 neuron current output, prevented further CaMKII-mediated enhancement of GluA1 homomeric AMPARs and halted FZP withdrawal-induced anxiety [13, 19, 21]. These findings contrast with the observed increase in NMDAR ligand binding and function following long-term treatment with the less selective positive allosteric GABA_A_R modulators, barbiturates and ethanol [45–47]. Instead, the observed reduction of NMDAR function and associated loss of postsynaptic CaMKII during benzodiazepine withdrawal could explain why the withdrawal syndrome associated with benzodiazepines is considerably less severe than withdrawal from barbiturates and ethanol [14, 48, 49].

## Figures and Tables

**Table 1:** *α*-CaMKII immunogold labeling.

		All synapses (≥0 particles)							≥1 particle in respective compartment			
		Bouton		Spine		PSD			Bouton	Spine	PSD	
Rat	Synapses sampled	Labeled (%) ≥1 particle	Number of particles	Labeled (%) ≥1 particle	Number of particles	Labeled (%) ≥1 particle	Particles/PSD length	PSD length (*μ*m)	Number of particles	Number of particles	Particles/PSD length	PSD length (*μ*m)
Control												
1	43	14.0	0.19	20.9	0.60	11.6	1.02	0.184	1.33	2.89	8.77	0.206
2	56	28.6	0.61	46.4	0.89	28.6	2.79	0.156	2.13	1.92	9.76	0.162
3	43	25.6	0.58	58.1	1.74	27.9	1.93	0.196	2.27	3.00	6.90	0.224
4	44	15.9	0.27	40.9	1.18	18.2	2.07	0.170	1.71	2.89	11.37	0.182
5	53	58.5	1.47	84.9	2.66	54.7	6.61	0.170	2.52	3.13	12.07	0.175

	Total 239											

Mean ± SEM		28.5 ± 8.0	0.62 ± 0.23	50.2 ± 10.6	1.41 ± 0.36	28.2 ± 7.3	2.88 ± 0.97	0.175 ± 0.007	1.99 ± 0.21	2.77 ± 0.22	9.77 ± 0.92	0.190 ± 0.011

FZP-withdrawn												
1	45	11.1	0.20	17.8	0.27	6.7	0.44	0.165	1.80	1.50	6.56	0.159
2	44	27.3	0.52	56.8	0.98	29.5	1.92	0.170	1.92	1.72	6.50	0.178
3	51	29.4	0.90	45.1	1.27	11.8	0.95	0.158	3.07	2.83	8.04	0.152
4	44	22.7	0.36	38.6	0.75	9.1	0.54	0.168	1.60	1.94	5.92	0.169
5	54	16.7	0.17	59.3	1.02	22.2	1.64	0.169	1.00	1.72	7.37	0.172

	Total 238											

Mean ± SEM		21.4 ± 3.4	0.43 ± 0.13	43.5 ± 7.5	0.86 ± 0.17	15.9 ± 4.3	1.10 ± 0.29	0.166 ± 0.002	1.88 ± 0.34	1.94 ± 0.23	6.88 ± 0.37	0.166 ± 0.005
*P* value		0.44	0.48	0.62	0.20	0.19	0.12	0.25	0.78	**0.03** ^ ∗^	**0.02** ^ ∗^	0.08

*P < 0.05, Student's *t*-test.

**Table 2:** pCaMKII immunogold labeling.

		All synapses (≥0 particles)							≥1 particle in respective compartment			
		Bouton		Spine		PSD			Bouton	Spine	PSD	
Rat	Synapses sampled	Labeled (%) ≥1 particle	Number of particles	Labeled (%) ≥1particle	Number of particles	Labeled (%) ≥1 particle	Particles/PSD length	PSD length (*μ*m)	Number of particles	Number of particles	Particles/PSD length	PSD length (*μ*m)
Control												
1	43	37.2	1.28	53.5	1.37	30.2	2.60	0.184	3.44	2.57	8.61	0.183
2	56	21.4	0.32	12.5	0.20	8.9	0.98	0.156	1.50	1.57	11.01	0.162
3	43	81.4	3.56	67.4	2.70	34.9	3.94	0.196	4.37	4.00	11.29	0.218
4	44	38.6	0.82	20.5	0.48	11.4	0.66	0.170	2.12	2.33	5.77	0.177
5	53	56.6	1.42	30.2	0.55	28.3	2.75	0.170	2.50	1.81	9.72	0.189

	Total 239											

Mean ± SEM		47.0 ± 10.2	1.48 ± 0.55	36.8 ± 10.3	1.06 ± 0.45	22.7 ± 5.3	2.19 ± 0.61	0.175 ± 0.007	2.79 ± 0.51	2.46 ± 0.43	9.28 ± 1.00	0.186 ± 0.009

FZP-withdrawn												
1	45	44.4	0.91	33.3	0.73	17.8	1.87	0.165	2.05	2.20	10.51	0.167
2	44	47.7	1.11	13.6	0.20	11.4	0.86	0.170	2.33	1.50	7.59	0.199
3	51	74.5	2.27	54.9	1.08	23.5	2.68	0.158	3.05	1.96	11.37	0.174
4	44	79.5	3.30	63.6	1.86	25.0	1.70	0.168	4.14	2.93	6.80	0.196
5	54	61.1	1.59	44.4	0.91	29.6	3.12	0.169	2.61	2.04	10.52	0.192

	Total 238											

Mean ± SEM		61.4 ± 7.0	1.83 ± 0.44	42.0 ± 8.7	0.96 ± 0.27	21.5 ± 1.3	2.05 ± 0.39	0.166 ± 0.002	2.84 ± 0.37	2.13 ± 0.23	9.36 ± 0.91	0.186 ± 0.006
*P* value		0.28	0.63	0.71	0.85	0.84	0.85	0.25	0.94	0.52	0.96	0.99

**Table 3:** CaMKII*α* and pCaMKII immunogold non-PSD synaptic labeling.

	Presynaptic						Postsynaptic					
	Active zone		Membrane		Intracellular		Perisynaptic membrane		Extrasynaptic membrane		Intracellular	
	Labeled (%) ≥1 particle	Mean Number of particles	Labeled (%) ≥1 particle	Mean Number of particles	Labeled (%) ≥1 particle	Mean Number of particles	Labeled (%) ≥1 particle	Mean Number of particles	Labeled (%) ≥1 particle	Mean Number of particles	Labeled (%) ≥1 particle	Mean Number of particles
CaMKII												
CON	4.0 ± 1.3	0.04 ± 0.01	3.0 ± 1.5	0.04 ± 0.02	26.2 ± 7.6	0.54 ± 0.20	0.8 ± 0.5	0.02 ± 0.01	3.9 ± 1.4	0.07 ± 0.04	38.8 ± 9.9	0.86 ± 0.22
FZP	0.8 ± 0.5	0.008 ± 0.005	2.6 ± 1.3	0.03 ± 0.01	19.8 ± 3.0	0.39 ± 0.12	2.6 ± 1.6	0.03 ± 0.02	4.8 ± 2.6	0.06 ± 0.04	32.2 ± 5.7	0.59 ± 0.12
*P* value	**0.049** ^ ∗^	**0.047** ^ ∗^	0.83	0.88	0.45	0.54	0.37	0.70	0.75	0.94	0.58	0.32

pCaMKII												
CON	7.2 ± 2.3	0.09 ± 0.03	4.5 ± 2.5	0.10 ± 0.08	43.7 ± 9.7	1.29 ± 0.46	0.9 ± 0.9	0.01 ± 0.01	2.3 ± 1.1	0.02 ± 0.01	21.9 ± 10.5	0.62 ± 0.34
FZP	7.3 ± 2.2	0.08 ± 0.03	6.7 ± 3.0	0.09 ± 0.04	58.6 ± 7.6	1.67 ± 0.40	3.1 ± 2.2	0.04 ± 0.03	5.1 ± 2.8	0.07 ± 0.05	26.9 ± 7.8	0.49 ± 0.18
*P* value	0.98	0.96	0.60	0.87	0.26	0.56	0.40	0.38	0.37	0.36	0.71	0.76

*P < 0.05, Student's *t*-test. Values represent mean ± SEM.

## Abbreviations

AMPAR: *α*-Amino-3-hydroxy-5-methyl-4-isoxazolepropionic acid receptor
CaM: Calmodulin
CaMKII: Ca/calmodulin-dependent protein kinase II
EDTA: Ethylenediaminetetraacetic acid
EGTA: Ethylene glycol tetraacetic acid
FZP: Flurazepam
GABA_A_R: *γ*-Aminobutyric acid type A receptor
L-VGCC: L-Type voltage-gated Ca channel
NMDAR: N-Methyl-D-aspartate receptor
PEG: Poly(ethylene) glycol
PSD: Postsynaptic density
TBST: Tris-buffered saline with 0.1% Tween 20.
